# The expanding universe of ribonucleoproteins: of novel RNA-binding proteins and unconventional interactions

**DOI:** 10.1007/s00424-016-1819-4

**Published:** 2016-05-10

**Authors:** Benedikt M. Beckmann, Alfredo Castello, Jan Medenbach

**Affiliations:** IRI for the Life Sciences and Institute for Biology, Humboldt-Universität zu Berlin, Philippstrasse 13, 10115 Berlin, Germany; Department of Biochemistry, University of Oxford, South Parks Road, Oxford, OX1 3QU UK; Institute of Biochemistry I, University of Regensburg, Universitaetsstrasse 31, 93053 Regensburg, Germany

**Keywords:** RNA-binding proteins, Protein-RNA interaction, Ribonucleoproteins, Interactome capture, RNA binding domain, RNA-determined RNPs

## Abstract

Post-transcriptional regulation of gene expression plays a critical role in almost all cellular processes. Regulation occurs mostly by RNA-binding proteins (RBPs) that recognise RNA elements and form ribonucleoproteins (RNPs) to control RNA metabolism from synthesis to decay. Recently, the repertoire of RBPs was significantly expanded owing to methodological advances such as RNA interactome capture. The newly identified RNA binders are involved in diverse biological processes and belong to a broad spectrum of protein families, many of them exhibiting enzymatic activities. This suggests the existence of an extensive crosstalk between RNA biology and other, in principle unrelated, cell functions such as intermediary metabolism. Unexpectedly, hundreds of new RBPs do not contain identifiable RNA-binding domains (RBDs), raising the question of how they interact with RNA. Despite the many functions that have been attributed to RNA, our understanding of RNPs is still mostly governed by a rather protein-centric view, leading to the idea that proteins have evolved to bind to and regulate RNA and not vice versa. However, RNPs formed by an RNA-driven interaction mechanism (*RNA-determined RNPs*) are abundant and offer an alternative explanation for the surprising lack of classical RBDs in many RNA-interacting proteins. Moreover, RNAs can act as scaffolds to orchestrate and organise protein networks and directly control their activity, suggesting that nucleic acids might play an important regulatory role in many cellular processes, including metabolism.

## The landscape of RNA-binding proteins

RNA assembles with proteins forming dynamic complexes named ribonucleoproteins (RNPs). The sequence of the transcript, its processing and the activity of the available RNA-binding proteins (RBPs) determine the composition of the RNP [42, 82], establishing an additional layer of information (the *RNP code*) that determines the fate of the RNA, shaping transcriptome and proteome. RNPs are not static, and their remodelling allows adjustments in gene expression under conditions that require adaptive changes. Dysfunction of RBPs is often linked to disease [19, 26, 28, 75, 88], which reflects the relevance of protein-RNA interactions in cellular homeostasis.

Given the complexity of RNA metabolism, it was estimated that RBPs comprise 3–11 % of the proteome of bacteria, archaea and eukaryotes and they are significantly more conserved across evolution than proteins lacking RNA-binding activity [3, 40]. Most of the RBPs discovered over the last three decades match the classical view of RBP architecture with a modular combination of well-characterised RNA-binding domains (RBDs) such as the RNA recognition motif (RRM), the K homology domain (KH) and the DEAD box helicase domain [76]. Individual RBDs recognise short stretches of RNA (approx. 2–10 nucleotides in length) with often low affinity. RBPs usually build their affinity and specificity for RNA on the cooperative function of multiple classical RBDs [49, 76], as exemplarily illustrated by the four RRMs that work together in nucleolin (NCL) or the poly(A)-binding protein (PABP).

A number of studies in the last two decades reported RNA-binding activities in proteins lacking classical RBDs, suggesting that the scope of RBPs was initially underestimated. These newly discovered RNA binders contain protein domains with dual function (e.g. enzymatic and RNA-binding activities) [114], folds of unknown function [54] and protein regions lacking a defined tertiary structure in the unbound state [95]. Given the growing evidence of unorthodox RNA-binding activities within proteins previously unrelated to RNA biology [24, 50, 86], system-wide approaches were developed aiming at determining the complete repertoire of RBPs.

In silico approaches were successfully used to identify proteins harbouring classical RBDs and highly homologous protein domains that may likely bind RNA as well [3, 40, 65]. However, the capacity of these methods to discover RNA-binding architectures that lack similarities with classic RBDs was very limited. To circumvent this, different in vitro and in vivo approaches for comprehensive identification of RBPs were developed. In two parallel studies, Scherrer et al. and Tsvetanova et al. used protein arrays to identify RBPs in *Saccharomyces cerevisiae*. A significant fraction of the yeast proteome (∼4000 proteins) was purified and immobilised on a membrane. The resulting array was subsequently incubated with a mixture of fluorophore-labelled RNAs, and fluorescence retained at each protein spot was used as readout for RNA binding. Using this method, these studies identified 180 [103] and 42 [111] proteins, respectively, as RBPs. Surprisingly, many of the identified proteins were not related to RNA biology, including dozens of metabolic enzymes such as oxidoreductases and proteins involved in lipid metabolism, calling for further in vivo validation of their RNA-binding activities.

**Fig. 1:**
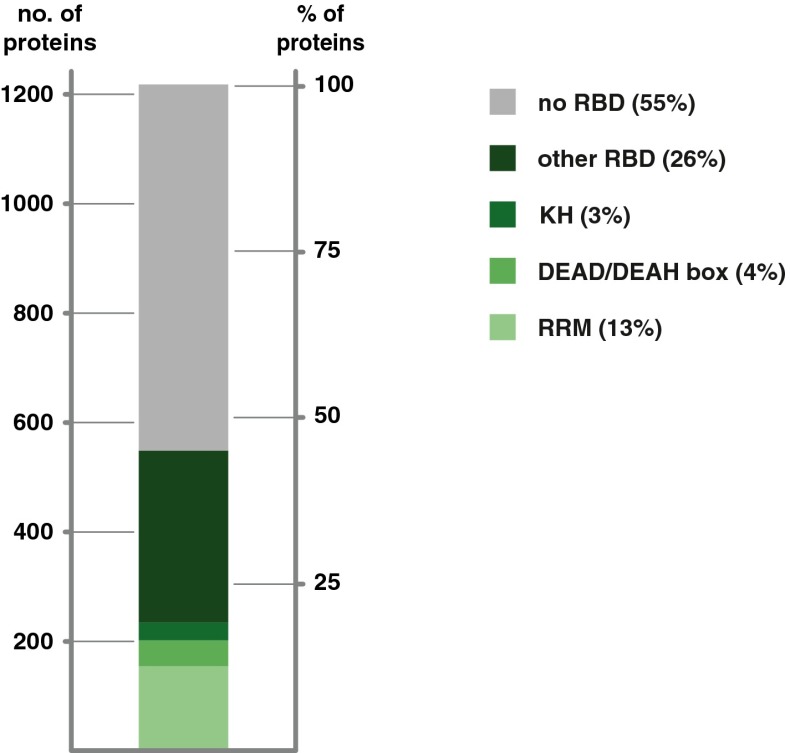
RNA interactome capture discovers many RNA-binding proteins that lack identifiable RNA-binding domains. RNA interactome capture from different human cell lines [8, 13, 18] identified a total of 1218 proteins as RNA binders, most of which do not contain an identifiable RBD (∼55 %). The remaining proteins harbour domains known to bind RNA, most commonly the RNA recognition motif (*RRM*, accounting for ∼13 % of the proteins), DEAD/DEAD box helicase domain (accounting for ∼4 %) and the K homology domain (*KH*, accounting for ∼3 %)

More recently, two independent works described a new method for the unbiased and comprehensive identification of RBPs from living cells, which is referred to as RNA interactome capture [8, 18]. In brief, native protein-RNA interactions are covalently immobilised by applying ultraviolet (UV) light to cultured cells [45, 52, 92]. UV irradiation induces short-lived free radicals at the nucleotide base that can attack amino acids in close proximity. Because proteins do not efficiently absorb UV light at these wavelengths, protein-protein cross-linking is not detectable [18, 107]. After irradiation, a stringent purification of polyadenylated (poly(A)) RNA is performed under denaturing conditions, followed by identification of co-purified, cross-linked proteins by quantitative mass spectrometry. The initial RNA interactome studies lead to the identification of 1106 RBPs in HeLa [18] and HEK293 [8] cells, with an extensive overlap between the two datasets (545 proteins). Both datasets do not only validate RNA binding of known RBPs, but they are also catalogued as novel RNA binders hundreds of proteins previously unrelated to RNA metabolism (561 proteins) [8, 18]. The newly identified RBPs belong to different protein families, participate in multiple biological processes, mediate distinct molecular functions and, surprisingly, in most of the cases, lack known RBDs (Fig. 1).

Subsequently, this methodology was extended to determine RNA interactomes in different cell lines and species. Kwon et al. added 283 novel RNA-binding proteins from mouse embryonic stem cells to the growing list of RBPs. Sixty-eight of these proteins are highly expressed in undifferentiated cells, suggesting a role in stem cell physiology [63]. Three independent studies identified 120 [81], 765 [78] and 678 [13] RNA binders in *S. cerevisiae*. Surprisingly, many enzymes from classical biochemical pathways, particularly from glycolysis, were found to moonlight as RNA-binding proteins in this unicellular organism. And finally, the interactome of an entire multicellular organism was derived from the worm *Caenorhabditis elegans*, identifying 594 poly(A) RNA-interacting proteins [78].

Comparison of the datasets revealed a conserved core of eukaryotic RNA-binding proteins, comprising ∼250 RBPs [13], most of which have known functions in RNA biology, e.g. in transcription, translation, RNA transport, degradation and/or modification. Surprisingly, around 9 % of the conserved RBPs also have reported enzymatic activities [13]. Presumably, datasets from additional species, including the most common eukaryotic model organisms (e.g. *Drosophila melanogaster*, *Danio rerio*, *Arabidopsis thaliana*) will become available soon, enabling further inter-species comparisons and even deeper insights into RNA biology.

## What sequences do the (novel) RBPs bind?

With the world of RBPs rapidly expanding and with more and more RBDs being identified, the need arises for a rapid identification of their binding motifs and target RNAs to gain insight into RBP function.

Numerous experimental methods have been developed to study protein-RNA interactions. These ribonomic analyses range from the determination of in vitro binding specificities of recombinant RNA-binding proteins (or domains thereof) to the purification and subsequent analysis of native RNPs. Employing a panel of recombinant and purified RBPs in single and competitive binding reactions against a complex pool of RNA ligands in vast excess (RNAcompete) has recently yielded the binding specificities of 207 RBPs from various species. Not surprisingly, it also revealed that two proteins that share a high degree of identity between their RBDs are likely to have similar or even identical RNA sequence specificity [98]. Another powerful method involves immunopurification of native RBPs from cell extracts and analysis of bound RNAs either by hybridisation to microarrays (RIP-Chip) or high-throughput sequencing (RIP-Seq) [84]. As described above for interactome capture, in vivo UV cross-linking has been employed to stabilise the highly dynamic environment of ribonucleoproteins and to preserve protein-RNA interactions through stringent affinity purification protocols, followed by the subsequent analysis of co-purified RNA (cross-linking and immunoprecipitation (CLIP), CRAC and related methods) [5]. The ‘freezing’ of protein-RNA interactions by UV cross-linking in living cells (or entire organisms) ensures that only interactions are captured that occur under native, cellular conditions. This is particularly important, as concerns were raised, that protein-RNA interactions which do not necessarily reflect the in vivo situation can occur after cell lysis in the extract [80, 100, 101].

Common to the aforementioned methods is that they do not only test for binary interactions of one protein (or RBD) with a single RNA species (or short oligonucleotide) but rather produce *global* or transcriptome-wide binding profiles. However, in most cases, studies are limited to individual RNA-binding proteins (or their respective RBDs). Expanding the analyses to other RBPs has been hampered either by the complexity of the experimental procedures, the availability of antibodies or the requirement for recombinant protein production.

Structural analyses of protein-RNA complexes have resulted in the identification of interaction surfaces and amino acids of proteins that are directly involved in RNA binding. This has significantly advanced our understanding of the principles that underlie binding specificity and RNP formation [6, 17]. Moreover, structures that contain multiple RBDs in complex with their ligand have unveiled how protein domains cooperate to recognise longer, continuous stretches of RNA to increase both RNA affinity and specificity [49]. However, our current structural knowledge of protein-RNA complexes is still rather limited. While the protein data bank (as of January 2016) lists more than 116,000 protein (or protein domain) structures, only less than 1.7 % (approx. 1800) are in complex with RNA. Moreover, the vast majority of the structural information on RNPs is derived from X-ray crystallography that requires rigid folds, such as globular protein domains. Hence, intrinsically unfolded proteins that bind RNA (see below) are highly underrepresented.

## Understanding RNA-binding specificity of proteins

Being able to predict the target RNA motif(s) based on the amino acid sequence of an RBD would greatly advance our understanding of RBPs and facilitate prediction of their cellular function(s), even if the proteins have not yet been extensively studied. However, the molecular basis of sequence-specific recognition of nucleic acids is well understood for only a subset of RNA-binding domains.

Pumilio and FBF homology (PUF) proteins employ a repetitive and modular scaffold for sequence-specific binding to RNA, each repeat recognising one nucleobase [116]. Breaking of this recognition code has allowed a rational design of custom proteins, tailored to recognise specific RNA sequences of interest, producing numerous genetically encoded tools to study RNA biology [117]. But despite the accumulating knowledge about RNP biology, understanding, predicting and engineering the specificity of RBDs other than the PUF domain remains a challenge. Hence, binding motif inference based on known specificities of closely related proteins still remains an approximation that requires experimental confirmation.

Moreover, RBPs do not necessarily only bind in a sequence-specific manner, recognising a stretch of specific nucleobases. The exon junction complex (EJC, its core comprising the proteins eukaryotic initiation factor (eIF)4AIII, Y14, Magoh and MLN51) has no apparent sequence specificity and is deposited ∼20 nucleotides upstream of exon-exon junctions by the splicing machinery. Its binding to the RNA is remarkably stable, allowing the EJC to stay associated with the RNA during nuclear export and requiring a dedicated disassembly factor in the cytoplasm [39, 64]. Other proteins recognise an RNA structure rather than a sequence or both. The protein Staufen 1 regulates messenger RNA (mRNA) localisation, stability and translation through binding to double-stranded RNA motifs of variable sequence. Analysis of its binding sites by CLIP has not only led to the identification of its RNA targets but also revealed thousands of RNA regions that form duplexes in vivo [99, 108]. Last but not least, a single protein (or protein complex) can recognise and bind to different RNA elements that are diverse in sequence and structure. This is exemplified by the RNA-binding protein She2p from *S. cerevisiae* that, together with its partner She3p, associates with different *cis*-acting RNA elements (so-called ‘zipcodes’) to mediate subcellular transport and localization of mRNAs [51, 87].

The complexity of protein-RNA interactions is probably best illustrated by the RNA recognition motif. Although being the most abundant and best-studied RBD, the RRM is also one of the most versatile. Despite a shared architecture, RRMs utilise different surfaces for RNA binding and display various different modes of interaction, thus making target prediction extremely difficult [6, 30]. Moreover, the RRM-type protein fold can also be employed for the interaction with proteins instead of RNA, giving rise to U2AF homology motifs (UHMs). These domains are highly similar to classical RRMs but lack some of the critical amino acids involved in RNA recognition, having instead evolved sequence characteristics that optimise the interaction with peptide ligands [60]. Interestingly, some RRMs can engage in both RNA and protein binding. This has been reported e.g. for the RRM domain of eIF3b, which associates with eIF3j or hepatitis C virus mRNA, presumably in a mutually exclusive manner [34, 93]. Another example is the RRM2 of the PABP that simultaneously interacts in a cooperative manner with the eIF4G and the poly(A) tail of the RNA to support translation initiation [102].

Taken together, this further complicates prediction of binding motifs and also highlights that in the cellular context, interactions between RBPs and their targets occur in a complex environment with many different binding partners available. Moreover, association with one ligand often impacts on interactions with other factors. Both mutually exclusive binding and highly synergistic binding have been reported for a number of RBPs. One example is the *Drosophila* RNA-binding protein Sex lethal (Sxl) which harbours two RRMs that bind U-rich sequences with high affinity. On the one hand, Sxl can compete with and evict other RNA-binding proteins or complexes such as U2AF, CstF64 and others [36, 37, 44], and on the other hand, it also acts as a nucleation factor to recruit proteins such as Upstream of N-ras (Unr) or Held-out wings (How) [1, 33, 44, 48]. This way, Sxl acts as a remodeler of RNPs, modulating their composition to control RNA fate.

Synergistic RNA binding of two proteins does not always necessitate a direct interaction between the two RBPs. Neither do antagonising factors always have to compete for binding to the same RNA element to exhibit a mutually exclusive binding behaviour. The RNA itself can relay the information of a binding event to a distal site through structural rearrangements. This can either improve accessibility or impede interaction with other factors. Exemplarily, the 3′ untranslated region (UTR) of vascular endothelial growth factor (VEGF) mRNA shows mutually exclusive and stimulus-dependent binding of two protein complexes, the HILDA (hypoxia-inducible hnRNP L-DRBP76-hnRNP A2/B1) complex and the interferon gamma (IFN-γ)-activated inhibitor of translation complex (GAIT). Binding of the HILDA complex results in a conformational change of the RNA that occludes the binding site of the GAIT complex. This creates a binary, molecular switch to adjust gene expression in response different stimuli [97, 124].

Despite the complexity of the interactions and experimental challenges to characterise RNPs, detailed insights into binding specificities and target RNAs have been obtained for numerous proteins [5]. This has revealed that in many cases, RBPs associate with mRNAs encoding functionally related proteins, forming the so-called ‘RNA operons’ [58]. Thus, a limited set of (or even individual) RBPs can control entire pathways through the coordinate regulation of functionally related transcripts, fine-tuning and adjusting gene expression to the cellular requirements.

In light of this, RNA interactome capture could well prove a treasure trove for RNA biologists. Experimentation can now be extended to proteins that previously had not been implicated in RNA biology but were identified as novel RNA binders by interactome capture, resulting in the discovery of novel RBDs. Exemplarily, interactome capture has contributed to the identification of the NHL domain (named after the NCL-1, HT2A and LIN-41 proteins) as a *bona fide* RBD. Several NHL domain-containing proteins were found to co-purify with RNA [8, 18, 63]. Subsequent biochemical and structural studies confirmed sequence-specific RNA-binding activity and discovered functions in post-transcriptional regulation of gene expression [63, 69–71].

Furthermore, identification of the RNA targets of newly discovered RBPs will provide insights into their cellular function and further advance our understanding of RNA metabolism. This is exemplified by the fas-activated serine/threonine kinase (FASTK) family. In humans, six FASTK protein family members are present and all of them co-purify with RNA in interactome profiling [18]. FASTK proteins harbour a RAP domain (RNA-binding domain abundant in apicomplexans) which exhibits a high degree of homology with the bacterial endonuclease-like fold and was predicted to bind RNA [18, 65]. Loss of the RAP domain in FASTKD2 is linked to mitochondrial cytochrome C oxidase-defective encephalomyopathy, a rare neurological disorder [41]. Recent work identified FASTKD2 as a component of mitochondrial RNA granules, implicating a function in mitochondrial RNA processing [4]. Finally, the identification of its target RNAs has paved the way for functional studies and established a link between FASTKD2 activity, mitochondrial dysfunction and human disease [96]. This example highlights how RNA interactome studies can guide experimentation in the discovery of unprecedented aspects of RNA biology.

In light of this, the newly identified RBPs and the growing number of novel RBDs that have been functionally validated by independent laboratories [13, 23, 38, 66, 88] predict a new age in RNA research with exciting discoveries to be made.

## RNA binding through low-complexity protein regions

Besides the abundance of proteins without a recognisable, classical RBD, large-scale datasets of RNA binders harbour another surprising finding: the identified proteins are enriched in low-complexity regions mainly composed of the amino acids serine (S), proline (P), glycine (G), arginine (R), lysine (K) and tyrosine (Y) [18]. These amino acids do not combine randomly but rather form defined patterns: G often co-occurs with R or Y, generating RG or YG repeats that can appear multiple times within a given protein region giving rise to highly repetitive sequences. Both serine and proline have a high propensity to be in disordered regions [68], and it has been predicted that many of the low-complexity regions in RBDs are intrinsically disordered regions (IDRs) which natively lack stable three-dimensional structure [16, 18, 46].

**Fig. 2:**
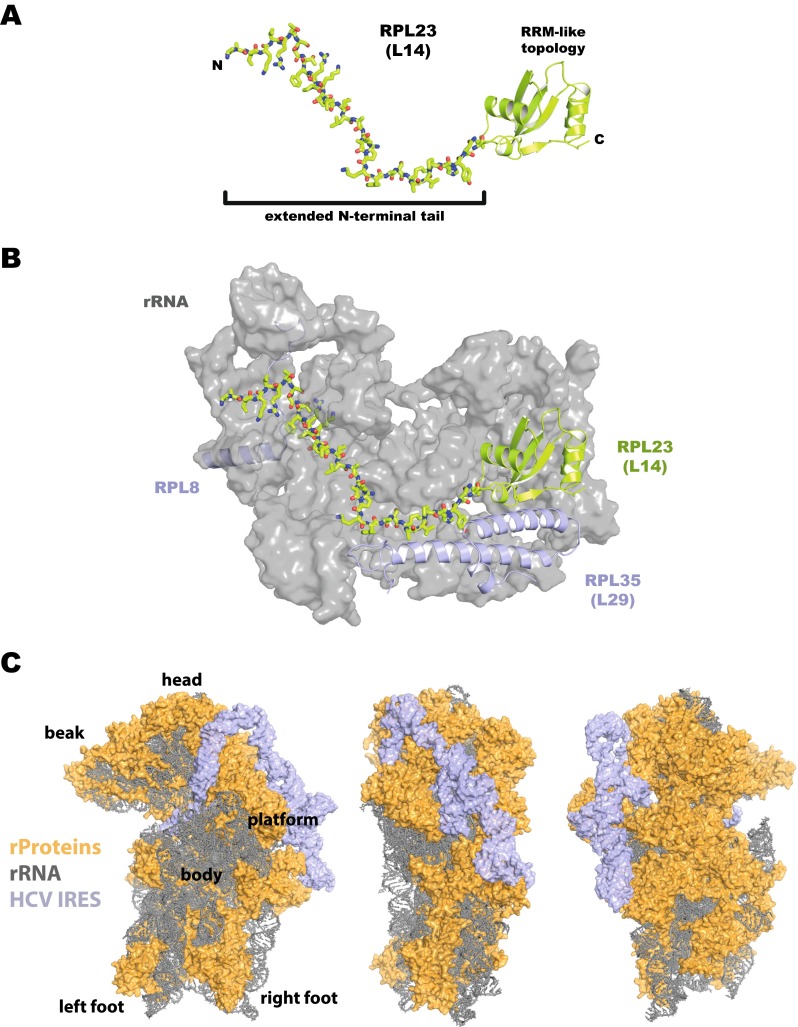
Protein-RNA interaction through low-complexity, extended protein regions and protein-binding RNAs. **a**, **b** The *S. cerevisiae* ribosomal protein L23 (*RPL23*) contains a C-terminal domain that folds into an RRM-like topology, whereas the N-terminal, low-complexity region adopts an idiosyncratic, extended conformation (**a**). In the 60S ribosomal subunit, the lysine- and arginine-rich N-terminal domain of RPL23 participates in extensive interactions with the ribosomal 25S and 5.8S RNAs (**b**). RNAs are depicted in *grey*, neighbouring proteins RPL8 and RPL35 in *light blue*; in the structure based on Ben-Shem et al. [14] (PDB ID: 4V88), residues within 20A of the RPL23 protein are depicted. **c** HCV IRES bound to a 40S ribosomal subunit. The HCV IRES (*blue*) displays an elongated structure that binds the solvent side of the 40S ribosomal subunit. Interactions are formed mostly with ribosomal proteins (eS1/S3A, uS7/S5, eS7/S7, uS11/S14, eS25/S25, eS26/S26, eS27/S27 and eS28/S28, proteins depicted in *yellow*) and, only to a lesser extent, with the ribosomal 18S RNA (shown in *grey*). In the structure based on Yamamoto et al. [123] (PDB ID: 5FLX), individual panels represent different orientations (rotated by 90°)

Interestingly, also ribosomal proteins often contain long extensions enriched in the amino acids G, R and K (Fig. 2a). While being flexible and often disordered in solution, these protein tails are found to adopt specific conformations in the ribosome, reaching deep into the core of the RNP and forming extensive interactions with the ribosomal RNA (Fig. 2b) [14, 15, 61]. Here, the flexibility and small size of G allows tight packing against the RNA, while the basic amino acids R and K contribute to RNA folding by mediating electrostatic interactions and neutralising the negative charge of the RNA backbone [61]. Similarly, unstructured *tails* rich in basic amino acids have been reported to play a role in protein-DNA interaction. By projecting into the minor grove of the DNA double strand, they increase local affinity and promote hopping and linear diffusion along the DNA molecule [112, 113]. And finally, a low-complexity, repetitive motif, the arginine-glycine-rich RGG box motif that is found in numerous RBPs [110], was demonstrated to interact with RNA [29]. In the fragile X mental retardation protein (FMRP), an RGG box binds with high affinity to guanidine-rich RNA sequences. This interaction is mediated by a combination of shape complementarity facilitated by the flexible G linker and the electrostatic potential of the arginines [95].

In addition to directly interacting with nucleic acids, IDRs in RNA-binding proteins have been shown to contribute to assembly and formation of RBP ultrastructures such as P bodies, stress granules or nuage [16]. They can directly promote quick phase transitions, resulting in the formation of droplets—membrane-free, dynamic cellular subcompartments that harbour specific RNPs [53, 89]. Acting as assembly domains, IDRs are capable of recruiting other IDR-containing proteins to these granules, enabling the formation of specialised compartments by phase transition. This molecular aggregation results in the formation of hydrogels and amyloid-like fibres, and misregulation of aggregation has been linked to several neurological disorders [47, 56, 67, 120].

Comparison of RNA interactome datasets from different organisms has revealed an expansion in the number of short linear motifs in RBPs in higher eukaryotes compared to unicellular yeast, while the number of classical RBDs remains similar [13]. In sum, the occurrence of IDRs might explain the RNA-binding properties of ∼100 novel human RBPs that lack globular RBDs [18].

## RNA-binding proteins and protein-binding RNAs

Surprisingly, interactome capture has also identified a number of proteins as RNA binders which do neither contain an identifiable globular RBD nor a predicted IDR. In human cell lines, more than half of the identified RBPs have no detectable RBD and have not been associated with functions in RNA metabolism before (Fig. 1) [8, 13, 18]. Similar results were obtained for rodents [63] and *C. elegans* [78], and an even larger fraction of proteins that lack identifiable RBDs were observed in yeast [13, 78, 81]. This raises an important question: are these proteins ‘false positives’ in interactome analyses, or do they contain novel (and yet unidentified) RBDs and/or bind RNA by non-conventional means?

Alike to proteins, also RNA can fold into intricate three-dimensional structures that serve various functions including catalytic activity, a classical example being the ribosome—a macromolecular and complex RNA machinery. Moreover, it has been demonstrated by systematic evolution of ligands by exponential enrichment (SELEX) that RNA sequences can be derived (so-called ‘RNA aptamers’) to selectively bind various different classes of small molecules [43]. Examples can also be found in nature: some bacterial mRNAs harbour sequences/structures with high affinity and specificity for various, structurally diverse metabolites. Ligand binding usually results in a conformational change of the RNA and in altered gene expression, forming a so-called ‘riboswitch’ that allows tailoring of protein production according to the nutritional status and cellular requirements [90].

Insofar, it is surprising that our understanding of RNPs is still mostly governed by a rather protein-centric view and the idea that proteins evolve to bind RNA and not vice versa. Often, it is difficult to differentiate between these two possibilities and there is clear evidence for co-evolution of proteins and RNA that interact to form an RNP. In fact, a continuous and densely populated spectrum exists, with one extreme being protein-determined RNPs (represented by many mRNPs) and the other one being RNA-determined RNPs like ribosomes [126]- the latter often considered relics, reminiscent of an RNA world in early evolution. In many RNA-determined RNPs such as ribosomes and other ribozymes, the enzymatic function is provided by the RNA, exposing it to much higher evolutionary pressure and often limiting the function of the bound proteins to being folding catalysts (RNA chaperones).

It is tempting to speculate that in RNA interactome datasets, the abundance of proteins that lack identifiable RBDs might, to some extent, reflect the capture of RNA-determined RNPs. In this case, RNAs have evolved to interact with proteins which hence do not require *canonical* RBDs.

An example of RNA-determined RNPs can be observed in a number of viral RNAs. Complex RNA secondary structures which promote viral translation can be found in the 5′ untranslated region of many different viruses, among them are picornaviruses, hepatitis C virus, herpes simplex virus and others. These so-called internal ribosome entry sites (IRESs) allow non-canonical and cap-independent translation initiation by functionally replacing some (or even all) eukaryotic translation initiation factors (eIFs), which are otherwise needed for *normal*, cap-driven translation [32]. Cap-dependent translation initiation is complex and requires the step-wise and hierarchical assembly of an RNP that then serves to recruit a small ribosomal subunit. By directly binding and stably recruiting specific eIFs (or even entire ribosomal subunits), viral IRESs can bypass some of the initial assembly steps, essentially becoming independent of initiation factors that act early. Exemplarily, the IRES of encephalomyocarditis virus binds directly to eIF4G, bypassing the requirement for eIF4E [72, 94], and the hepatitis C virus IRES interacts directly with the eIF3 complex and small ribosomal subunits (Fig. 2c) [74, 105, 109]. The binding of HCV IRES to eIF3 requires a certain geometry of the RNA and involves specific bases at critical positions [59], and structural studies have revealed extensive interactions between the IRES and eIF3, covering a broad surface area [104]. Moreover, an association of the HCV IRES with small ribosomal subunits employs mostly interactions with ribosomal proteins and only, to a lesser extent, interactions with the 18S ribosomal RNA (Fig. 2c) [123]. Even though classical RNA-binding domains of protein interaction partners appear to be involved to some extent, the interactions with the IRES involve surface areas of the proteins that differ from the ones that are usually employed for canonical interactions [93]. In sum, this supports the notion that the viral RNA evolved to specifically bind cellular proteins to support viral translation. IRES activity has also been reported for some cellular RNAs; however, factor requirements and interactions are not nearly as well characterised as for their viral counterparts [35, 121].

What appears to be a common theme to aptamers and IRES elements is that ligand/protein binding requires (extensive) RNA structure elements. While in rare cases promoting internal translation initiation, extensive RNA structures in 5′ UTRs or even coding regions of mRNAs are otherwise inhibitory to translation. Hence, mRNAs whose major role is to serve as templates for protein synthesis are less likely to contain such intricate structural elements that function in protein binding. However, for non-coding RNAs, these constraints do not apply and one might envision that RNA aptamers that bind proteins might be more common in this class of RNAs. In fact, many of the well-studied non-coding RNAs form extensive secondary structures to associate with proteins. This includes several classes of abundant RNAs such as small nuclear RNAs (snRNAs) involved in splicing [122], small nucleolar RNAs (snoRNAs) that direct RNA modification [119], the RNA moiety of the signal recognition particle (SRP RNA or 7SL RNA) critical for membrane protein insertion and protein secretion [73], RNase P RNA essential for transfer RNA (tRNA) processing [83] and telomerase RNA involved in chromosome maintenance [127].

## Functional and structural roles of RNA

The function of RNA extends well beyond the mere coding for peptides. Exemplarily, RNA is not only the major structural component of ribosomes but also contributes catalytic activity and delivers amino acids for protein synthesis. Moreover, RNAs play integral roles in RNA processing and modification, are involved in sensing of metabolites and act as scaffolds to organise larger complexes. Several classes of small non-coding RNAs provide specificity for proteins, acting as guides. In eukaryotes, microRNAs (miRNAs), small interfering RNAs (siRNAs) and Piwi-associated RNAs (piRNAs) assemble with proteins of the argonaute clade and recruit them to their nucleic acid targets by (partial) sequence complementarity, eliciting gene regulatory pathways [79]. Similarly, snoRNAs and the closely related small Cajal body-specific RNAs (scaRNAs) direct RNA modification, determining targets through base paring interactions [119]. In prokaryotes, clustered regularly interspaced short palindromic repeat (CRISPR)-Cas immune systems rely on RNAs to identify foreign nucleic acids, targeting them for degradation [77]. Here, CRISPR RNAs (crRNAs) and trans-encoded crRNAs (tracrRNAs) serve as guides for Cas nucleases to direct DNA cleavage [55]. Harnessing the power of Cas nucleases and their RNA guides has revolutionised genome editing in many organisms [22, 55, 77, 106].

In telomerase, the RNA moiety does not only serve as a template for synthesis of telomeric repeats by the telomerase reverse transcriptase (TERT) but also provides a flexible scaffold to assemble the entire RNP, providing binding platforms for the Ku heterodimer, the LSm heteroheptameric protein complex and TERT [126].

Recently, long non-coding RNAs (lncRNAs), a heterogeneous and functionally diverse group of transcripts in eukaryotes, have gained widespread attention. They are polyadenylated, often contain extensive secondary structure and elicit different functions, serving e.g. as scaffolds, decoys and/or guides [62, 115]. Exemplarily, the formation of paraspeckles, a nuclear compartment rich in factors with roles in RNA processing, depends on the lncRNA nuclear-enriched autosomal transcript 1 (NEAT1). The RNA plays an architectural role, interacting with and nucleating several proteins such as paraspeckle protein 1 (PSP1); splicing factor, proline- and glutamine-rich (SFPQ) protein; and p54nrb/NONO (non-POU-domain-containing octamer-binding protein). Knockdown of the RNA results in a loss of paraspeckles and redistribution of proteins [25], reflecting the importance of the RNA for the spatial organisation of this compartment.

## RNA as a regulator of protein activity

The structural role of RNA in the organisation of RNPs, where it e.g. serves as a (flexible) scaffold, is well documented, and its function as guide for proteins, directing them to their sites/sequences of action, is also well established. But can RNAs also regulate the activity of their bound protein partners?

Some of the best-documented examples, highlighting the function of RNAs as regulators of protein activity, are found in innate immunity. Toll-like receptors involved in the pattern recognition of pathogens are activated by double-stranded RNA (TLR-3), single-stranded RNA (TLR-7/8) and bacterial ribosomal RNA (TLR-13) [57, 91, 125]. The activity of the cytoplasmic retinoic acid-inducible gene I (RIG-I)-like receptors, nucleotide oligomerization domain (NOD)-like receptors and interferon-induced proteins with tetratricopeptide repeats (IFITS) depends on recognition of RNAs with 5′-triphosphate structures [2, 9]. And finally, protein kinase R (PKR) is activated by long double-stranded RNA (dsRNA) which is often generated during viral replication. dsRNA can trigger PKR dimerisation resulting in autophosphorylation and activation [7, 27]. Among the PKR targets is the eIF2α that plays critical role in translation initiation, escorting the initiator tRNA to the small ribosomal subunit. Phosphorylation of eIF2α by PKR traps it in an inactive state [31], impairing cellular translation and viral replication.

But RNA regulation of protein activity is neither restricted to innate immunity nor eukaryotes. Bacterial 6S RNA is a ∼200-nt-long, non-coding RNA which inhibits transcription of housekeeping genes in *Escherichia coli* and *Bacillus subtilis*. It adopts a rod-shaped secondary structure with a flexible central region, thereby mimicking an open promoter to bind and inhibit the bacterial RNA polymerase [118]. 6S RNA is one of the most abundant RNA species in bacterial cells during stationary growth phase and was identified in all branches of the bacterial kingdom [11]. While the sequence of this ncRNA varies widely between species, its secondary structure is predicted to be highly conserved among prokaryotes as well as its binding to RNA polymerase and its inhibitory activity [12]. Other RNAs that directly interact with the transcription machinery to regulate gene expression have also been identified in eukaryotic organisms and viruses [10].

In light of RNA being a regulator of protein function, it is intriguing that many of the proteins that are found to associate with RNA are reported to exhibit enzymatic activities. Dozens of enzymes from intermediary metabolism associate with RNA, including almost all glycolytic enzymes [13, 21, 85, 86]. A particularly interesting example is glyceraldehyde-3-phosphate dehydrogenase (GAPDH), a key enzyme in glycolysis that has recently been reported to interact with the 3′ UTR of the IFN-γ mRNA, inhibiting its translation [21]. The RNA-binding activity of GAPDH depends on the glycolytic activity of the cell: upon activation, the metabolism of T lymphocytes shifts from oxidative phosphorylation to aerobic glycolysis, preventing the association of GAPDH with RNA and allowing IFN-γ translation [21]. Similarly, cellular iron metabolism controls the function of the iron regulatory protein (IRP) 1. In iron-deficient cells, IRP1 binds to and regulates translation and stability of various RNAs; however, in the presence of iron, it assembles an iron sulfur cluster (4Fe-4S) and functions as a cytoplasmic aconitase [20, 85, 114]. GAPDH and IRP1 present two interesting examples of mutually exclusive regulation: RNA binding is incompatible with enzymatic activity and protein function switches from metabolism to post-transcriptional regulation of gene expression.

Taken together, RNA is more than a mere bystander molecule that is being regulated by proteins. Various RNAs can play active roles in the organisation of ribonucleoproteins, sometimes even shaping entire subcellular compartments. Moreover, RNAs can control the function and activity of their bound protein partners, adjusting cell physiology to changing cellular requirements. In sum, this has fuelled the hypothesis that RNA may orchestrate enzymatic activities by bringing together all the enzymes that are part of a given metabolic pathway into large protein assemblies, forming metabolons with superior metabolic performance [20].

Scaffolding, protein binding and regulation of enzymatic activity have, however, only been described for a limited set of RNAs. The large number of *unconventional* RNA-binding proteins that were recently discovered might be a hint that many more examples of *functionally active* RNAs are waiting to be discovered. Characterisation and functional studies of RNPs that contain novel RNA-binding proteins with enzymatic activities will pave the way for a better understanding of how RNA biology integrates with other, in principle unrelated, cell functions such as intermediary metabolism.
